# Error-corrected ultradeep next-generation sequencing for detection of clonal haematopoiesis and haematological neoplasms – sensitivity, specificity and accuracy

**DOI:** 10.1371/journal.pone.0318300

**Published:** 2025-02-26

**Authors:** Melinda L. Tursky, Crisbel M. Artuz, Melissa Rapadas, Gary A. Wittert, Timothy J. Molloy, David D. Ma

**Affiliations:** 1 Blood, Stem Cell, and Cancer Research Programme, St Vincent’s Centre for Applied Medical Research and Department of Haematology, St Vincent’s Hospital, Sydney, Australia; 2 School of Clinical Medicine, St Vincent’s Healthcare Clinical Campus, Faculty of Medicine and Health, UNSW Sydney, Kensington, Australia; 3 Freemasons Centre for Male Health and Well-Being, South Australian Health and Medical Research Institute and Faculty of Health and Medical Sciences, University of Adelaide, Adelaide, South Australia, Australia; European Institute of Oncology, ITALY

## Abstract

Clonal haematopoiesis of indeterminate potential (CHIP) is an aging-associated phenomenon that has recently been correlated with a broad spectrum of human diseases, including haematological malignancy, cytopenia, coronary heart disease, stroke, and overall mortality. CHIP is defined as a somatic variant in blood cells with an allele frequency (VAF) ≥ 0.02, however recent reports show smaller clones are associated with poorer clinical outcome. Error-corrected ultradeep next-generation sequencing (NGS) assays detecting variants < 0.02 VAF also have clinical value for monitoring measurable residual disease (MRD) for myeloid neoplasms. However, limited data are available on optimal parameters, limits of detection, and accuracy of ultra-sensitive detection. We investigated parameters to improve accuracy of Illumina sequencing-by-synthesis method, including read depth, input DNA quantity, and molecular barcoding-based data filtering, while adhering to clinical accreditation criteria. Validation data were generated from reference standards and reference samples from a clinically accredited pathology laboratory. Analytical range measurements included linearity and bias, and precision included repeatability, reproducibility and detection rate. The lower limit of detection was ≥ 0.004 (0.4%) at depth > 3,000 × . Trueness measured using reference standards demonstrated a sensitivity, specificity, positive and negative predictive values, and accuracy of 100%, including FLT3-ITD, and 100% concordance was achieved with reference samples for reported variants and absence of variants. Sequencing blood samples from 383 community-dwelling adults (mean depth 3758×) revealed 2,190 somatic variants/sample, > 99.9% were < 0.02 VAF. Our data including cost-benefit analysis enables pathology and research laboratories to make informed decisions for detection of CHIP (VAF ≥ 0.02), sub-CHIP (VAF 0.01–0.02) and MRD (VAF ≥ 0.004).

## Introduction

Haematopoietic stem and progenitor cells can acquire somatic mutations due to exogenous or endogenous stressors or a loss of fidelity in DNA replication [1]. The progeny of these cells form a clonal sub-population sharing the mutational landscape of the founder cell. Some variants may confer competitive advantages such as increased survival or self-renewal resulting in increased frequency. This accumulation of somatic mutations during human aging leading to clonal blood cell expansion is known as clonal haematopoiesis [2]. Somatic mutations in blood at ≥ 0.02 variant allele frequency (VAF) in individuals without overt haematological disease is commonly termed Clonal Haematopoiesis of Indeterminate Potential (CHIP) [3]. This VAF threshold is largely arbitrary however, and partly based on the limit of detection in early whole genome, whole exome, and targeted next-generation sequencing (NGS) CHIP studies that used low-to-moderate sequencing depths (most <<1,000×).

The most commonly mutated genes observed in CHIP, including *DNMT3A*, *TET2*, and *ASXL1*, are also recurrent in myeloid malignancies. A key distinction being that large clones (VAF > 0.035) are almost always mutually exclusive in CHIP but usually co-occur in myeloid malignancies [4]. These observations led to the hypothesis that CHIP was a pre-leukaemic disorder, however recent studies demonstrated that CHIP can persist long-term without progression to overt haematological disease and importantly is associated with a gamut of other disorders including stroke, cytopenia, heart disease, metabolic and neurodegenerative disorders, infection, and overall mortality in adults [4–7].

The decreasing cost of sequencing coupled with innovations such as unique molecular identifiers (UMI) for error-corrected sequencing, which decrease error rates from 0.005-0.02 to ≥ 0.0001, significantly increased the sensitivity and specificity of somatic variant detection [8,9]. Some recent studies used high-depth targeted NGS to demonstrate that presence of somatic variants below the CHIP threshold of 0.02 VAF are predictive of all-cause mortality and may be associated with development and progression of several diseases common with aging [10–12]. These findings suggest reliable CHIP detection at VAF lower than the traditional threshold may have clinical value.

Ultra-high sensitivity NGS platforms are also used for measurable residual disease (MRD) monitoring in haematological malignancies such as acute myeloid leukaemia (AML) [13,14]. Techniques for MRD detection are less prone to reproducibility issues when a single or small number of high-confidence/pre-validated variants are selected for longitudinal monitoring [14]. In studies of CHIP however, the use of NGS to survey large genomic regions to detect small clones that may not have been independently validated may have significant clinical value. While the validation of germline variant detection and large somatic mutation detection has been comprehensively discussed in the literature and are routinely used in clinical NGS laboratories, the precision and accuracy of ultra-high-depth NGS detection for clones of < 0.02 VAF requires additional investigation. Herein we aimed to evaluate the utility of a targeted 75-gene myeloid-focussed NGS assay using anchored multiplex PCR-based error-corrected sequencing for ultra-sensitive detection of CHIP in blood. The presented data suggest an optimal target for mapped read depth, input DNA quantity, and data filtering to balance sequencing cost (directly proportional to depth) with the reproducible detection of CHIP variants down to 0.004 VAF.

## Materials and methods

### Subjects and samples

Patient and healthy subject samples were collected with written informed consent (approved by St Vincent’s Hospital Human Research Ethics Committee, Australia, HREC 13/SVH/26, recruitment start 12 March 2013 and ongoing). Blood samples from 383 community-dwelling adults were used to investigate CHIP prevalence. These were collected as part of the Florey Adelaide Male Aging Study (FAMAS) [15–17], a longitudinal study examining health outcomes of community dwelling men aged 35–80 years living in north-west of Adelaide, Australia (approved by Royal Adelaide Hospital Research Ethics Committee and, where appropriate, the Aboriginal Health Research Ethics Committee of South Australia).

### Targeted next-gen sequencing assay

The VariantPlex Myeloid (Invitae, Colorado, USA) targeted NGS panel was used, which utilises anchored multiplex PCR for molecular barcoded, strand-specific library generation for 75 gene targets (S1 Table; total target size 125.4kb). Library preparation was undertaken per manufacturer’s instructions using 50–400ng of input DNA. Genomic DNA concentrations were determined using the Qubit dsDNA HS Assay Kit with the Qubit 3 Fluorometer (ThermoFisher Scientific, Massachusetts, USA). Individual library concentrations were quantified using the KAPA Library Quantification Kit (Kapa Biosystems) and libraries were pooled and requantified for sequencing. NGS was performed on Illumina (California, USA) NextSeq 500 and NovaSeq 6000 sequencers at a targeted depth of 3,000–5,000x.

### Known reference standards, controls, and blood sample sequencing

The Myeloid DNA (HD829) and True-Q 100% Wildtype (HD752) Reference Standards (Horizon Discovery, Cambridge, United Kingdom) were used. These contain DNA from multiple cell lines, with substitutions, indels, and duplications (including FLT3-ITD) at known VAF from 0.05–0.70. Eight independent libraries were prepared using serial dilutions of HD829 with mononuclear cell-derived DNA from a healthy 24-year-old male subject to generate variant panels of accessible variants with known VAF ranging from 0.0008-0.40. HD752 contains variants and wildtype versions of several genes. Additionally, DNA from peripheral blood or bone marrow mononuclear cells of 16 patients with variants and 3 participants without detectable variants as identified by a clinically accredited pathology laboratory (reference samples) were used to determine concordance for variants per the reference laboratory lower limit of detection (LLOD) (S2–S4 Tables). Genomic DNA was isolated using the QIAamp DNA Blood mini kit (Qiagen, California, USA), DNA purity was determined by spectrophotometry and concentration by Qubit assay. The clinicopathological characteristics of study subjects are summarised in S5 Table.

### Droplet digital PCR (ddPCR)

Custom TaqMan VIC/FAM-labelled ddPCR assays (ThermoFisher Scientific, Massachusetts, USA) were run per manufacturer’s instructions on the QX200 Droplet Digital PCR System (Bio-Rad, California, USA). ddPCR reaction mixes were prepared with template gDNAs, Supermix (Bio-Rad) and TaqMan primer-probes (S6 Table) and partitioned into oil droplets (~20,000) by the QX200 droplet generator.

### Bioinformatic and statistical analysis

**Fig 1:**
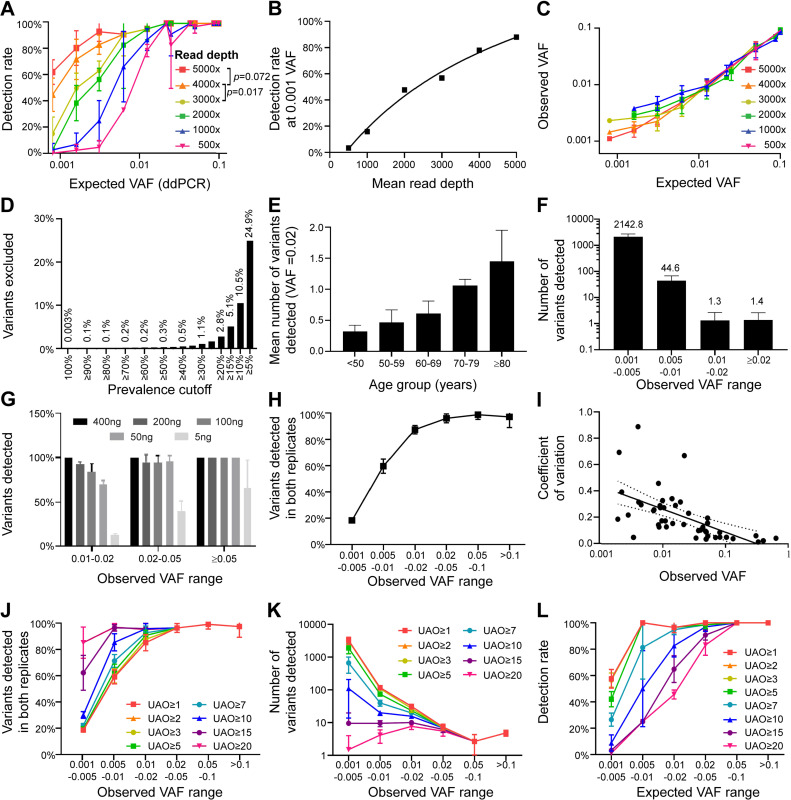
Optimisation of read depth, DNA quantity, and unique alternate observation threshold. A: The detection rate of reference standard variants ranging from 0.0008 to 0.1 VAF at 500-5,000 × depth. Variants ≥ 0.02 VAF could be detected at 97% as low as 500 × , whereas 5,000 × was required to detect two-thirds of variants at 0.0008 VAF. A target depth of 2,000-3,000 × detected 95% of variants ≥~0.01 VAF. B: The relationship between the detection rate of very small (~0.001 VAF) variants and sequencing depth. A target depth of 3,000-4,000x detected 60-80% of variants at ≥~0.001 VAF. C: The association between expected VAF and observed VAF according to read depth. Lower read depth may contribute to an overestimated VAF of very small variants, although differences did not reach significance. D-F: Blood samples from 383 community-dwelling subjects were sequenced at a target depth of 3,000-4,000 × . Variants with a prevalence of ≥ 10% within this cohort were excluded as likely sequencing artefacts, which excluded 10.5% of variants (D). The mean number of variants detected per sample above the standard CHIP threshold of ≥ 0.02 VAF increased linearly with subject age (E). Greater than 99.9% of variants detected were below ≥ 0.02 VAF, potentially including those with prognostic or predictive clinical relevance (F). G: Libraries were prepared with 5–400ng of input DNA. Using somatic variants detected in the 400ng sample as “ground truth”, the mean detection rate was determined for VAF ranges with 0.01–0.02, 0.02–0.05, and ≥ 0.05. Almost all variants at ≥ 0.02 VAF were detected using an input DNA of ≥ 50ng, however detection of variants at < 0.02 VAF benefitted from increasing input DNA amounts. H: The detection rate of variants detected in samples from 4 AML patients from which duplicate library preparations were performed. Across both independent replicates 96% of variants were detected at VAF ≥ 0.02, 85% for 0.01–0.02 VAF, 60% of variants at 0.005-0.01 VAF, and 18% of variants at 0.001–0.005 VAF. I: The CV of variant size quantitation across independent replicates, which displayed a strong negative correlation to variant size (dotted lines indicate 95% confidence interval). J-L: Increasing unique alternative observation (UAO) thresholds for variant calls increased detection rate of rare variants (J), but markedly decreased the number of variants passing the threshold (K) and therefore reduced detection of true positive variants in reference standards (L). Error bars represent standard deviation.

Paired-end reads (150 bp) were de-multiplexed into individual FASTQ files and aligned to the Hg19 human reference genome, and variant calling was undertaken using Archer Analysis 6.0.1 software (Invitae). This identified single nucleotide polymorphisms (SNPs), insertions and deletions (indels), and performed FLT3-ITD detection. Variants were filtered to identify missense, nonsense, frameshift, splice site, insertion, and deletion mutations. A variant was classified as likely pathogenic if it was a frameshift or nonsense mutation in a coding region. Mutations were excluded if they had ≥ 5% prevalence in the gnomAD database [18] of variants from individuals across the global population; the allele frequency was < 0.0001; strand bias was detected (statistically significant probability (p ≤ 0.05) of asymmetry in the positive vs. negative strands as determined by primers binding to each (and indicative of deamination or PCR error)); were likely germline variants (VAF 0.45-0.55, or ≥ 0.95); and/or the mutation detected was not predicted to be functionally relevant. Functionally relevant variants were defined as located in the 5’ UTR or coding sequence, and/or resulting in feature elongation/truncation, frameshift, inframe deletion/insertion, missense mutation, protein altering variant, start/stop codon loss, transcript ablation, transcript amplification, splice acceptor variant, splice donor variant, and/or splice region variant. Following optimisation (Fig 1), a UAO filter of ≥ 3 was applied, specifying the minimum number of input DNA molecules required for variant calling. In addition, for FAMAS blood samples, any variant with a prevalence > 10% of samples was excluded as a likely sequencing artefact. Data analysis was performed using Prism 9 (GraphPad) and PASW Statistics 18 (SPSS). All data was confirmed to meet the assumptions of statistical tests used. Quantitative data are presented as mean ±  standard deviation (SD).

## Results

### Optimisation: sequencing depth versus variant detection rate

We aimed to experimentally validate the sequencing depth required to detect variants at given frequencies under real-world conditions, focusing on detection of mutations below the CHIP threshold (0.02 VAF). Horizon HD829 Myeloid DNA Reference Standards were serially diluted in leukocyte-derived DNA from a healthy 24-year-old subject, yielding samples with assessable mutations in 15 genes at known VAF ranging from 0.0008–0.40. Three independent libraries were prepared from each concentration using the VariantPlex Myeloid targeted NGS library preparation kit and sequenced to a target depth of 5,000 × . Assay detection rates at lower depths were determined by random subsampling of reads (read depth normalisation). Detection was successful for 97% of variants (24 variants in 18 genes for all 3 independent replicates) ≥ 0.02 VAF at a depth of ≥ 500 × (Fig 1A).

Recent studies indicate clones at 0.005-0.02 VAF also have prognostic value [10–12]. Here reproducible detection of mutations of < 0.02 VAF rapidly diminished at lower depths, with variants at ≤ 0.001 VAF unable to be detected at depths ≤ 2,000 × . Ultra-high-depth sequencing 5,000 × detected a mean 63% of variants at 0.0008 VAF (Fig 1A). The mean detection rate of mutations at ≤ 0.005 and ≤ 0.001 VAF was significantly higher at depth 4,000 × than 3,000 × (*p* =  0.009 and 0.017 respectively), however their detection rate at 5,000 × was not significantly better than 4,000 × (*p* =  0.122 and 0.072 respectively), as assessed by t-test. Sequencing at increased depth is associated with increased cost and time (S7,S8 Tables). These observations combined with diminishing returns of increasing depth, particularly at VAF > 0.01, suggest the optimal balance between cost, time and detection rate for CHIP may be 2,000–3,000x, at which approximately 95% of variants near 0.01 VAF were detected (Fig 1A), and 3,000–4,000x for VAF near 0.001 with 60–80% of variants successfully and reproducibly detected (Fig 1B). In addition to reduced detection at low VAF, low read depth may also influence the accuracy of observed VAF. Analysis showed a trend toward an overestimation of observed VAF with ≤ 2,000x depth for ≤ 0.003 VAF, although this did not reach statistical significance (Fig 1C).

We used 3,000–4,000 × target depth to determine how many somatic variants could be detected in the blood of 383 community-dwelling males (S5 Table). Bioinformatic filtering removed variants with high prevalence in the global population (previously reported in the gnomAD database at ≥ 5% prevalence), those that exhibited strand bias (p ≤ 0.05), had low allele frequency (VAF < 0.0001), and/or variants not predicted to be functionally relevant (see Materials and Methods). This resulted in 93,354 mutations with VAF ≥ 0.001 observed in the 125.4kb surveyed across the cohort. Of these, an additional 95 variants that exhibited a VAF of 0.45–0.55 or ≥ 0.95 were removed as suspected germline variants. A second prevalence filter was also applied under the assumption that highly recurrent mutations within this community-dwelling cohort (prevalence ≥ 10%) were more likely to be PCR error or other sequencing artefacts than true mutations, which removed an additional 10,961 putative mutations (10.5% of those remaining after bioinformatic filtering). The use of such prevalence filters is common [10,19,20], however, the cutoff selection is largely arbitrary. The impact of prevalence cutoff on the number of variants excluded in the cohort data is indicated in Fig 1D. A mean 1.4 variants (range 0–7) were found per subject at ≥ 0.02 VAF, which increased with subject age (Fig 1E). At the lower threshold of > 0.001 VAF, a mean 2,190 (range 951–3954; Fig 1E) somatic variants were observed per individual, meaning 99.9% of detected variants were below the commonly used CHIP VAF threshold. This suggests many somatic mutations, some of potential clinical relevance, have been excluded from previous CHIP studies that used less sensitive sequencing methodologies.

### Optimisation: input DNA amount versus variant detection rate

Most NGS library preparation protocols support a wide range of DNA input amounts, with targeted sequencing panels often recommending 10–250ng. We investigated the influence of input DNA amounts on variant detection using 5ng, 50ng, 100ng, 200ng, and 400ng of input DNA derived from blood mononuclear cells of three healthy, FAMAS males (aged 56-61 years). Using somatic variants detected in the 400ng sample as “ground truth”, the mean detection rate was determined at various VAF. Greater than 94% of variants with ≥ 0.02 VAF were detected using ≥ 50ng of input DNA; 39% of variants with 0.02-0.05 VAF, and 65% of variants with ≥ 0.05 VAF using 5ng of input DNA (Fig 1G). Sub-CHIP (0.01–0.02 VAF) variant detection was more dependent on input DNA, with ≥ 100ng of input DNA required to detect ≥ 84% of variants, and 5ng of input detecting only 12%. This suggested CHIP-level variants can reliably be detected in samples where DNA is limiting, but where material is available and/or sub-CHIP variant detection is required, the upper limit of input DNA should be used.

### Optimisation: unique alternate observation threshold vs rare variant detection

The detection of unexpectedly large numbers of < 0.01 VAF clones raises the question what proportion represent true clones or, for example, library preparation or sequencing artefacts or isolated remnants of degraded DNA. To investigate this, we determined the detection rate of rare variant detection using AML patients. Independent duplicate NGS libraries were prepared from 4 AML patients at diagnosis and sequenced to a mean depth of 3420 × . The mean detection rate of all variants across both duplicates was determined at various VAF (Fig 1H). At VAF ≥ 0.02 96% of variants were detected across both replicates. This decreased to 85% at 0.01-0.02 VAF, 60% at 0.005-0.01 VAF, and 18% for variants at 0.001-0.005 VAF. Concordant with poor rates of detection at very low VAFs, the variance of clone size quantitation increased significantly at lower VAF (Fig 1I). Clones at VAF ≥ 0.02 had a mean coefficient of variance (CV) of 0.11, versus 0.24 for clones between 0.01-0.02, and 0.32 for clones < 0.01. When accurate quantitation of small clones is important such as in MRD tracking, where an increase in clone size is associated with poor outcome [14], increased depth and/or input DNA is therefore recommended.

The poor detection rates of very rare clones suggested that reliability of detection events at this level may be low. We therefore investigated whether detection rate could be improved bioinformatically, by filtering data using a Unique Alternate Observation (UAO) threshold. UAO takes advantage of molecular barcoding during library preparation and describes the number of unique DNA molecules in which a given variant appears during variant calling. Applying increased UAO stringency markedly increased the detection rate of rare variants, for example a UAO of ≥ 20 resulted in 98% of variants being detected in both replicates at 0.001-0.005 VAF (Fig 1J). Increased UAO stringency significantly limited the number of variants passing the filter however, with a mean 1.5 variants at 0.001-0.005 VAF with UAO ≥ 20 versus 3174 with UAO ≥ 3 (Fig 1K). To determine what proportion of real variants were being excluded at increasing UAO values, the detection rate for serially-diluted reference standards was determined (Fig 1L). UAO ≥ 10 effectively precluded detection of almost all variants at VAF ≤ 0.005, suggesting these stringent UAO requirements decreased sensitivity too far for reliable detection of rare variants. Detection rates were largely constant using a UAO of ≤ 3 (detecting 97% of variants at VAF ≥ 0.005, ~ 65% at a VAF of 0.0031 and ~ 51% at VAF 0.0016), and steadily decreased from UAO ≥ 5 which successfully detected 49% and 35% of variants at these VAFs. The UAO of ≥ 3 therefore provides the optimal threshold to maintain high detection rates while minimising false negatives. Table 1 provides a summary of the expected VAF (manufacturer supplied, ddPCR) and average observed VAF for reference standard HD829 undiluted and at various dilutions, using the optimised parameters.

**Table 1:** Expected and observed VAF, read depth and UAO for reference standard variants. Reference standard HD829 was serially diluted with leukocyte-derived DNA from a healthy 24-year-old subject. Undiluted (100%) and diluted samples (25%, 6.25%, 3.13% and 1.56%) were used to compare expected VAF (manufacturer’s supplied, ddPCR) to observed VAF for 15 accessible variants, using the optimised parameters illustrated in Fig 1. ND, not detected.

Panel concentration:			100%				25%				6.25%				3.13%				1.56%			
Gene	Amino Acid Change	Variant Type	Expected VAF (ddPCR)	Observed VAF (NGS)	Depth x	UAO	Expected VAF (ddPCR)	Observed VAF (NGS)	Depth x	UAO	Expected VAF (ddPCR)	Observed VAF (NGS)	Depth x	UAO	Expected VAF (ddPCR)	Observed VAF (NGS)	Depth x	UAO	Expected VAF (ddPCR)	Observed VAF (NGS)	Depth x	UAO
ABL1	T315I	SNP	0.0500	0.0481	1433	47	0.0125	0.0093	1395	12	0.0031	0.0046	1463	7	0.0016	0.0029	1723	5	0.0008	ND	ND	ND
ASXL1	G646fs*12	INS	0.4000	0.3099	6287	445	0.1000	0.0815	6995	216	0.0250	0.0411	7215	148	0.0125	0.0337	7611	132	0.0063	ND	ND	ND
ASXL1	W796C	SNP	0.0500	0.0487	2993	86	0.0125	0.0110	2813	25	0.0031	0.0034	2274	7	0.0016	ND	ND	ND	0.0008	ND	ND	ND
CBL	S403F	SNP	0.0500	0.0540	1571	63	0.0125	0.0099	1781	17	0.0031	0.0047	2395	11	0.0016	0.0029	2115	6	0.0008	ND	ND	ND
FLT3	D835Y	SNP	0.0500	0.0144	4429	32	0.0125	0.0034	5443	15	0.0031	0.0012	5057	5	0.0016	ND	ND	ND	0.0008	ND	ND	ND
GATA2	G200Vfs*18	DEL	0.3500	0.3372	1962	216	0.0875	0.0687	1694	72	0.0219	0.0166	1429	19	0.0109	0.0073	1686	11	0.0055	0.0055	1566	8
IDH1	R132C	SNP	0.0500	0.0407	3765	91	0.0125	0.0085	4855	33	0.0031	0.0033	5240	16	0.0016	0.0017	6219	10	0.0008	0.0014	4241	6
JAK2	F537-K539>L	DEL	0.0500	0.0442	1558	43	0.0125	0.0101	2148	17	0.0031	0.0037	2381	7	0.0016	ND	ND	ND	0.0008	ND	ND	ND
JAK2	V617F	SNP	0.0500	0.0502	2444	83	0.0125	0.0112	2969	28	0.0031	0.0029	3170	9	0.0016	ND	ND	ND	0.0008	ND	ND	ND
KRAS	G13D	SNP	0.4000	0.4036	1612	213	0.1000	0.0819	2074	90	0.0250	0.0158	1727	16	0.0125	0.0073	2449	15	0.0063	0.0032	2352	8
NPM1	W288Cfs*12	INS	0.0500	0.0262	1342	30	0.0125	0.0084	1402	11	0.0031	ND	ND	ND	0.0016	ND	ND	ND	0.0008	ND	ND	ND
NRAS	Q61L	SNP	0.1000	0.1029	1688	66	0.0250	0.0215	2092	31	0.0063	0.0058	1413	6	0.0031	0.0027	2454	6	0.0016	ND	ND	ND
SF3B1	G740E	SNP	0.0500	0.0477	4572	123	0.0125	0.0086	5951	43	0.0031	0.0024	3255	6	0.0016	0.0014	3930	10	0.0008	0.0011	5430	6
TET2	R1261H	SNP	0.0500	0.0476	1112	36	0.0125	0.0100	1325	12	0.0031	0.0045	1308	6	0.0016	0.0038	1583	5	0.0008	ND	ND	ND
TP53	S241F	SNP	0.0500	0.0510	3242	93	0.0125	0.0095	3595	32	0.0031	0.0022	1738	7	0.0016	0.0018	4326	7	0.0008	0.0015	4008	6

### Analytical range

**Fig 2:**
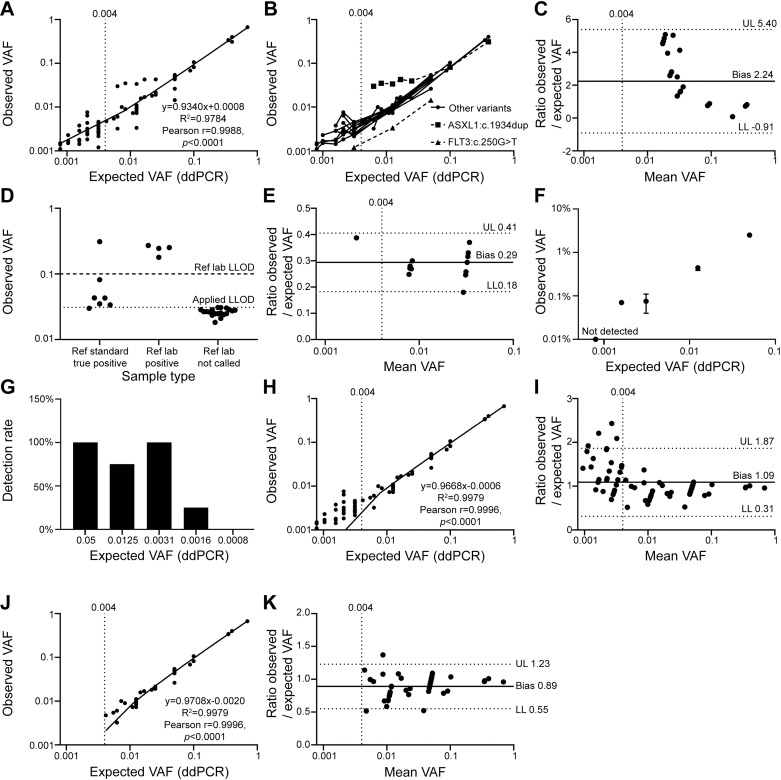
Analytical range of DNA reference standards. **A-**B: Overall, Horizon reference standards with an average read depth of 3121x showed linearity (R^2^ = 0.9784) across dilutions with a strong Pearson correlation (*p* < 0.0001) to manufacturer’s expected VAF (ddPCR to an average read depth of 582x) (A). **C.** Assessment of individual variants identified an over-estimation of ASXL1:c.1934dup at VAF ≤ 0.025. **D**. The lower limit of detection (LLOD) for ASXL1:c.1934dup was set at 0.031 VAF, which is below the VAF of reference standard and reference sample true positives and above observed VAF in reference samples not called by the reference laboratory. **E.** Variant FLT3:c.2503G > T showed consistent negative bias of 0.3-fold compared to expected VAF. **F**-G: FLT3-ITD showed a linear relationship across dilutions (F), and a 100% detection rate was observed as low as 0.0031% VAF at a mean depth of 3128 × , though this decreased rapidly at lower VAFs (G). H-I: Variants other than ASXL1:c.1934dup and FLT3:c.2503G > T showed a strong linear relationship (R2 = 0.9979) and Pearson correlation (p < 0.0001, H), with a positive bias at VAF ≤ 0.004 (I). J-K: Limiting VAF to ≥ 0.004 revealed a bias of 0.89-fold. Error bars represent standard deviation. Vertical dotted line indicates 0.004 VAF. Horizonal solid and dotted lines in Bland-Altman plots (C, E, I, K) indicate bias, and the upper limit (UL) and lower limit (LL) of 95% confidence intervals.

Reference standard dilutions used with optimised parameters showed linearity (R^2^ = 0.9784) with a strong Pearson correlation (r = 0.9988, *p* < 0.0001) between expected and observed VAF (Fig 2A). Assessment of individual variants (Fig 2B) identified an over-estimation in observed VAF for ASXL1:c.1934dup from VAF ≤ 0.043 (Fig 2B-C ). Sequencing issues of ASXL1:c.1934dup are well documented due to its location in an 8 base-pair mononucleotide guanine (G) nucleotide repeat, which can cause false positive artefacts and increased repeat length [21], and a separate LLOD for this variant was used by the reference laboratory (S2 Table). Herein LLOD for ASXL1:c.1934dup was set at 0.031 VAF, as this was below reference standard and reference sample true positives, and above the detected levels in reference samples not called by the reference laboratory (Fig 2D). FLT3:c.2503G > T displayed a consistent negative bias of 0.3-fold (Fig 2B,D) across reference standards, libraries and sequencing machines (included in Fig 2E) potentially due to sequencing or bioinformatic issues [22]. Dilutions of the reference standard showed a linear relationship of FLT3-ITD (Fig 2F), with 100% detection rate at VAF ≥ 0.0031, with rapid reduction at lower VAF (Fig 2G). Exclusion of ASXL1:c.1934dup and FLT3:c.2503G > T, due to variant-specific bias, enabled more precise analysis of linearity and revealed a positive bias for variants < 0.004 VAF (Fig 2H,I). Excluding variants with VAF < 0.004 (Fig 2J,K) revealed an overall bias of 0.9-fold when comparing expected VAF (manufacturer determined by ddPCR at an average depth of 582x) to observed VAF ≥ 0.004 (average depth 3121×).

### Precision

**Fig 3:**
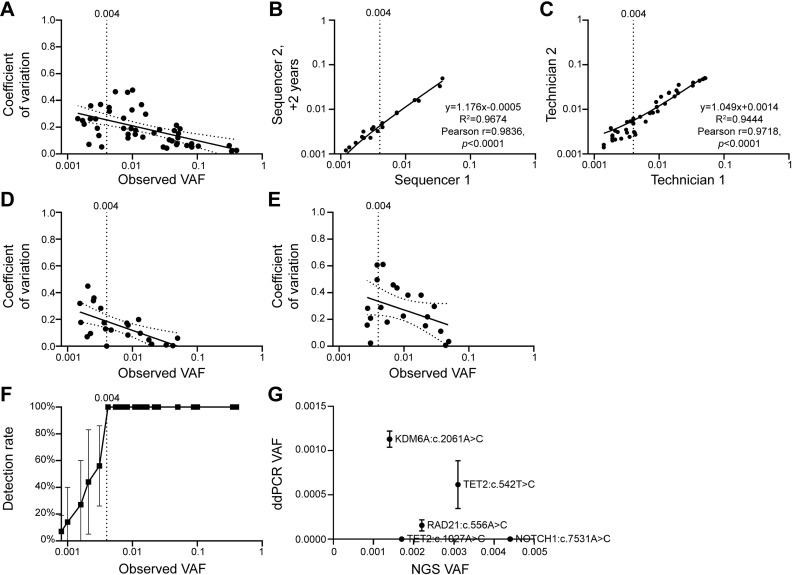
Precision and lower limit of detection. A: Repeatability of optimised assay showing intra-technologist and -sequencer machine variability as VAF vs CV. Average CV increased with decreased VAF. B-C: Reproducibility inter-sequencer machine (B) and inter-technologist (C) demonstrated high linearity (R2 = 0.9674, 0.9444) and Pearson correlation (both p < 0.0001). D-E: CV varied according to technologist, and consistently showed higher CV with decreasing VAF. F: Detection rate of the optimised assay was 100% for the reference standards at VAF ≥ 0.004 but fell rapidly < 0.004 VAF. LLOD was therefore set at VAF ≥ 0.004. G: The detection of five rare clones (<0.005 VAF) identified in blood samples from community-dwelling subjects sequenced at a mean depth of 3485 × with a UAO filter of ≥ 3 were independently validated by ultra-sensitive ddPCR. Of these, 3 (60%) were successfully detected, consistent with the moderate potential false-positivity rate at < 0.005 VAF. Error bars represent standard deviation. Vertical dotted line mark the LLOD of 0.004 VAF. Dotted lines on CV indicate 95% confidence interval.

Repeatability of detection of reference standards was high across intra-run and inter-run repeats, and demonstrated a negative correlation between CV and VAF (Fig 3A). The observed mean CV of 0.10 at VAF 0.05 is comparable to the CV of 0.102 in the reference laboratory using a target depth of 500x (S2 Table). Mean CV of 0.20 was observed at a VAF of 0.01–0.02, and 0.26 at a VAF 0.004-0.01. Reproducibility was also high between different sequencer machines utilised 2 years apart (Fig 3B) and different technologists (Fig 3C), showing a strong linearity (R^2^ = 0.9674, 0.9444) and Pearson correlation (r = 0.9836, 0.9718, both *p* < 0.0001). CV of two staff consistently showed increased variability with reduced VAF (Fig 3D,E).

Detection rate in the clinical pathology setting defines the LLOD as the VAF at which ≥ 95% of replicates are detected [23,24]. The detection rate of all DNA reference standards was 100% across 8 independent library preparations with VAF ≥ 0.004 (and ASXL1:c.1934dup at VAF ≥ 0.031) but dropped to 56% at VAF 0.0031 (Fig 3F). The LLOD for this assay was therefore VAF ≥ 0.004 for all except ASXL1:c.1934dup.

The reduced detection rates of reference standards (resulting in false negatives) at VAF < 0.004 (Fig 3F), highlights the possibility of false positives detected in subject samples at similarly low VAF, perhaps arising from sequencing or library preparation artefacts. To investigate, ddPCR was used as an independent, non-NGS-based method for verification to quantify five ≤ 0.005 VAF mutations that were found in more than 1 individual from the targeted sequencing of blood samples from community-dwelling adults (S6 Table, Fig 3G). Three (60%) were detected by ddPCR, with 2 undetectable across 3 independent technical replicates. Given the exquisitely high sensitivity of ddPCR, with a reported ability to reproducibly detect variants at ≤ 0.00005 VAF [25], the two variants that remained undetectable by this method are likely to be false positive NGS detection events.

### Trueness

Validation of assays for clinical pathology laboratories includes multiple measures of trueness [23,24]. Herein trueness was determined with an LLOD of ≥ 0.004 VAF for all variants (ASXL1:c.1934dup LLOD of 0.031 VAF). Reference standards HD829 and HD752 were used to determine the detection of true positives (TP, 23 variants), false positives (FP), false negatives (FN) and true negatives (TN, wild type sequences of 29 variants). Clinical samples from 16 reference laboratory samples were used to determine concordance of 36 variants reported as above clinical LLOD (S2–S4 Tables). Results showed a 100% concordance between 59 true positive variants from reference standards and samples. Sensitivity, specificity, positive predictive value, negative predictive value, and accuracy were all calculated to be 100% (Table 2), which exceeds clinically acceptable criteria [23,24]. Assessment of True Negative value using clinical samples is complex as an absence of reads may result from insufficient read depth and more correctly be a False Negative [26]. Designation of clinical samples as Presumed Negative was applied when they were negative for all tested variants in the accredited pathology laboratory. Three such samples were used to identify variants detectable using our optimised ultra-deep NGS (Table 3). A total of 193 variants were detected. However, 108 of these were not covered by the reference laboratory panel, and the remaining 85 variants were covered but detected below the reference laboratory LLOD. There was, therefore, 100% concordance for absence of variants with all three Presumed Negative samples.

**Table 2:** Reference standard and clinical sample measures of trueness. Trueness validation of optimised assay with a lower limit of detection (LLOD) of ≥ 0.004 VAF (and LLOD 0.031 for ASXL1:c.1934) using reference standards HD829 and HD752 true positives (TP, 23 assessed variants) and true negatives (TN, wild type sequences of 29 assessed variants), and clinical samples from a reference laboratory included 16 participants to determine concordance of 36 variants reported above the reference laboratory LLOD and 3 participants without detectable variants (Presumed Negative clinical samples) to determine concordance for absence of variants. Results showed a 100% concordance between 59 true positive variants and 29 true negatives (concordance for absence of variants). Sensitivity, specificity, positive predictive value, negative predictive value and accuracy were determined to be 100%, which exceed the acceptable criteria of ≥ 98%.

Measures		Reference standards	Clinical samples	Total
**Concordant variants**	True Positive (TP)	23/23 100%	36/36 100%	59/59 100%
**Called but not positive in reference standards or clinical samples**	False positive (FP)	0%	0%^*^	0%
**Not called**	False negative (FN)	0%	0%	0%
**Concordance for absence of variants**	True negative (TN)	29/29 100%	100%^*^	29/29 100%
**Measures**		**Reference standards** **and clinical samples**		**Acceptable** **criteria**
**Sensitivity %**	TP/ (TP + FN)	100%		≥98%
**Specificity %**	TN/ (FP + TN)	100%		≥98%
**Positive predictive value**	TP/ (TP + FP)	100%		≥98%
**Negative predictive value**	TN (TN + FN)	100%		≥98%
**Accuracy**	(TP + TN)/ (TP + FN + TN + FP)	100%		≥98%

* Assessment of True Negative and False Positive using clinical samples is complex, refer to main text and Table 3.

**Table 3:** Detect ion of variants in presumed negative clinical samples. A total of 193 variants were detected in Presumed Negative reference laboratory samples. However, 108 of these were not covered in the reference laboratory panels, and the remaining 85 variants were detected below the reference laboratory LLOD at the time of testing. Hence 100% concordance for absence of variants was achieved.

Category	# Individual variants
Total # Variants detected ≥ 0.004 VAF in Presumed Negative clinical samples*	193
# Variants not covered in reference laboratory panel	108
# Variants covered but below reference laboratory LLOD	85
# Remaining variants called in Presumed Negative clinical samples	0
Concordance for absence of variants within the reference laboratory panel and reference laboratory LLOD	100%
* Presumed Negative clinical samples had no variants called across all tested variants in an accredited pathology laboratory.	

### Utility

Table 4 summarises the utility of the VariantPlex Myeloid targeted 75-gene NGS panel using anchored multiplex PCR-based error-corrected sequencing for the detection of CHIP (VAF ≥ 0.02), sub-CHIP (VAF 0.01-0.02) and MRD (VAF ≥ 0.004). Included is a cost-benefit analysis and recommendations regarding read depth, DNA quantity, and data filtering for each VAF target range.

**Table 4:** Utility of optimised assay with a lower limit of detection of ≥ 0.004. For a target VAF range the optimal read depth and DNA quantity is indicated to achieve 100% accuracy. Indicated relative total cost and time include raw data file size for 144 samples and the cost for data storage, cost and time for library preparation and quantification, sequencing, and bioinformatic analysis (refer to S7–S8 Tables). Also included are the number (mean and range) of somatic variants observed in community dwelling individuals, and the coefficient of variance (CV) in reference standard and community dwellers at each VAF range.

VAF target	Optimal depth	Relative total cost	Relative total time	Optimal DNA quantity (ng)*	Optimal UAO level	Sensitivity and Specificity	Positive / negative predictive values	Accuracy	Variants in community dwellers (mean (range))	CV of reference standards	CV of community dwellers
≥0.02	1,000 –2,000x	0.8x	0.8x	≥50	≥1	100%/100%	100%/100%	100%	1.4 (0–7)	0.1	0.11
**0.01–0.02**	**2,000** **–3,000x**	**1x**	**1x**	**200–400**	**≥3**	**100%/100%**	**100%/100%**	**100%**	**1.3** **(0–9)**	**0.2**	**0.24**
0.004–0.01	3,000 –4,000x	1.1x	1.1x	400	≥3	100%/100%	100%/100%	100%	154.2 (24–440)	0.26	0.32

* If available the upper limit of input DNA should be routinely used.

## Discussion

Clinical studies of haematological malignancies have demonstrated the value of CHIP and MRD detection in diagnosis, prognosis, and management [27]. Recent reports of CHIP below 0.02 VAF have also demonstrated clinical relevance across multiple disease groups [10–12,28] in adults unselected for haematological disease. Both the European LeukemiaNet MRD Working Party and the United States Prescribing Information include NGS detection thresholds down to 0.0001 VAF for MRD risk prediction “if confidently detected above background noise” [29,30].

Due to increasing demand for NGS assays to detect small genetic variants in clinical settings, we evaluated the utility of detection of small clones ( ≤ 0.02 VAF) in blood by error-corrected ultra-high-depth targeted NGS of the sequencing-by synthesis method [29]. Balancing cost, sensitivity and reproducibility, our data indicate 400ng input DNA, a target depth of 3,000-4,000 × , and a UAO filter of ≥ 3 is recommended for low VAF variant detection with the NGS panel and platform used (see Table 4). Clinical accreditation criteria [23,24,31] were employed for validation of analytical range (linearity and bias), precision (repeatability and reproducibility), and determined the LLOD to be 0.004, with ASXL1:c.1934dup ascribed an LLOD of 0.031 VAF due to its location. Validation of trueness demonstrated sensitivity, specificity, positive and negative predictive values, and accuracy of 100%, which exceeded accreditation requirements [23,24,31]. This included FLT3-ITD, which is below levels normally thought to be clinically relevant [32]. Detection of very small clones (<0.005 VAF) should be confirmed with an orthogonal method such as ddPCR to validate ultra-rare variants of interest.

Multiple factors influence the limits of detection and sequencing error in NGS technologies. Error-corrected ultra-deep sequencing increases sensitivity but quickly approaches the LLOD at sub-CHIP VAF since a library preparation utilising 100ng of input DNA will on average only represent ~ 15 diploid cells bearing a variant of interest at 0.001 VAF [33]. Use of stringent UAO filters, that specify the minimum number of required input DNA molecules, reduced detection rates by filtering out ultra-rare variants. This can be optimised by increasing input DNA quantity, as demonstrated here and in another study, thereby raising the number of reads per variant [34]. Where input DNA is limited such as clinical samples, the depth and filters recommended here may be appropriate.

Errors introduced by the imperfect fidelity of polymerases, template switching, hairpin formation during library preparation, or errors in data capture during the sequencing process can result in false positives [35]. Additionally, pooling libraries, as in the current study, may introduce error in calling low VAF variants, with a bias towards false positives [36]. The complexity of the genomic target regions under interrogation can further affect sequence accuracy and sensitivity [37]. With library preparation and sequencing fidelity likely difficult to improve significantly further, and the benefits of library pooling on cost efficiency outweighing the increased error rate for most cases, improved bioinformatic methods including optimised error correction systems may be the most fertile area of research to further reduce these confounders [38,39].

The data and recommendations presented herein adhere to clinical accreditation criteria. They are also supported by the few previous studies that investigated the limits of detection of targeted NGS assays for MRD to sub-CHIP (0.001-0.02 VAF) clones. In MRD detection in AML Balagopal *et al.* demonstrated 94.9% specificity at > 0.001 VAF using ultra-high-depth (>10,000x), 400ng input DNA, and UMI barcoding [40]. We achieved 100% specificity to ≥ 0.004 VAF using less depth (3,000-4,000x) and 400ng DNA. Our analysis showed greater depth identifies more variants but at greater cost, and reduced specificity and reproducibility. At sub-CHIP (≥0.01 VAF) Min *et al.* demonstrated 100% detection of reference standard variants with a mean depth of 2,191 × [41]. This matches our recommended depth of 2,000-3,000x for 0.01-0.02 VAF with 100% accuracy across reference standards and clinical samples. For CHIP (≥0.02 VAF) Shin *et al*. used experimental and simulated data and achieved 95% sensitivity and positive predictive value at 1,085 × depth [20], this matches our recommended depth 1,000-2,000x for ≥ 0.02 VAF. Kluk *et al.* observed increased false positivity rate and reduced reproducibility at VAF < 0.05 [40]. Our data demonstrated no false positives and 100% detection to VAF ≥ 0.004, using depth 3,000-4,000x, 400ng DNA, and UAO ≥ 3. These studies support our data and recommendations for detection of variants at various target VAF (Table 4).

Limitations of this study include clinical sample availability from the reference laboratory, with 16 samples used as true positive samples and 3 lacking any detectable variants used as presumed negative samples. Ideally a minimum of 59 clinical samples would ensure 95% confidence intervals regardless of data being parametric or nonparametric [23]. This study used a single NGS panel on the sequencing-by-synthesis platform, which may be suited to probing different genomic features than the Ion semiconductor sequencing platform [42].

The observation that CHIP has widely been associated with a broad spectrum of chronic diseases yet very few specific mutations have been demonstrated as disease drivers suggests that these small clones may yet be shown to play an important clinical role. NGS studies with improved accuracy and reproducibility will be crucial to investigating this possibility and will increase the capability for concurrent MRD longitudinal monitoring of larger numbers of variants with high confidence. Our cost-benefit analysis and recommendations (Table 4) will further facilitate clinical pathology and research laboratories to make informed decisions about detection of CHIP (VAF ≥ 0.02), sub-CHIP (VAF 0.01-0.02) and MRD (VAF ≥ 0.004). High accuracy and detection rate at VAF ≥ 0.004 is achievable with investment of 1.1-fold higher total cost and time than detection of CHIP and sub-CHIP variants at ≥ 0.01 VAF.

## Supporting information

S1 Table Gene coverage of the Invitae VariantPlex Myeloid targeted NGS assay. Shown are variant type, accession number and exons detected. SNV single nucleotide variant, indel insertion or deletion, CVN copy number variant and/or internal tandem duplication (ITD).
(PDF)

S2 Table Reference laboratory samples and variants. Grouped as those with reported variants and those without, followed by participant diagnosis, showing the number of participants per group, number of assessable genes on each panel, number of variants reported by the reference laboratory, lower limit of detection (LLOD) used at the time of reference laboratory testing, number of samples negative for all variants tested by the reference laboratory. AML acute myeloid leukaemia, MDS myelodysplastic syndrome, MPN myeloproliferative neoplasm. * At the time testing by the reference laboratory the panels covering 7 or 26 genes used a 0.05-0.10 LLOD at a target read depth of 1000x for the majority of variants, an LLOD of 0.01 for JAK2:c.1849G > T and KIT:c.2447A > T and assessed ASXL1:c.1934dup by fragment analysis with an LLOD of 0.10. **The reference laboratory panel covering 41 genes used an LLOD of 0.02 and a target read depth of 500x for the most variants, with CEBPA LLOD ~ 0.10, ASXL1:c.1934dup LLOD ~ 0.05, and a stated coefficient of variance (CV) at 0.05 VAF of 0.102.
(PDF)

S3 Table Observed variants matching Reference laboratory reported variants. Grouped by diagnosis, showing variant, average observed VAF, depth and UAO. AML acute myeloid leukaemia, MDS myelodysplastic syndrome, MPN myeloproliferative neoplasm.
(PDF)

S4 Table Reference laboratory panels. Showing assessable genes.
(PDF)

S5 Table Clinical characteristics of 383 community-dwelling adult males. Part of FAMAS cohort, including any previous diagnosis of Type II Diabetes, cancer, ischemic heart disease, or stroke, and prevalence of clonal haematopoiesis at different clone sizes.
(PDF)

S6 Table Custom TaqMan Assays (ThermoFisher Scientific) for droplet digital PCR (ddPCR). Used to independently validate low VAF CHIP variants identified from a cohort of community-dwelling adults.
(PDF)

S7 Table Impact of target read depth on cost and time shown relative to values for a depth of 3000x. Comparison of parameters to sequence 144 samples at specific read depths of 500x, 1000x, 2000x, 3000x, 4000x and 5000x, shown relative to values for a depth of 3000x (indicated in bold). Time for bioinformatic analysis covers the pipeline from raw NGS data to output of a list of variants and does not include curation of this list. Lower read depths (500x) can be achieved by including a higher number of samples per run. This required the same cost and time per 144 samples for preparation and quantification, and resulted in an 0.8-fold lower total cost and 0.7 less total time due to the increased number of samples per run and smaller raw data files. Increasing depth, to enable accurate sequencing of very low VAF resulted in a 1.1 to 1.3-fold higher total cost and time requirement overall. Refer to S8 Table for original values for this assay in hours and Australian dollars.
(PDF)

S8 Table Comparison of cost and time parameters to sequence 144 samples at specific read depths. Raw data file sizes are in gigabytes (GB), costs are in Australian dollars (AUD) and time is indicated in hours. Time for bioinformatic analysis covers the pipeline from raw NGS data to output of a list of variants and does not include curation of this list. Lower read depths (500x) can be achieved by including higher number of samples per run, and results in smaller raw data file size per 144 samples. The cost and time for preparation and quantification per 144 samples remains the same. Cost of sequencing, data storage, time for sequencing and bioinformatics all vary by raw data file size, resulting in changes to total cost and time. Refer to S7 Table for these values shown relative to values at a read depth of 3000x (indicated in bold).
(PDF)
