# Supplementary material for: Error-corrected ultradeep next-generation sequencing for detection of clonal haematopoiesis and haematological neoplasms – sensitivity, specificity and accuracy
Source: PLoS One. 2025 Feb 26;20(2):e0318300. doi: 10.1371/journal.pone.0318300 (PMC11864513; doi:10.1371/journal.pone.0318300)
Supplement: S1 Table — Shown are variant type, accession number and exons detected. SNV single nucleotide variant, indel insertion or deletion, CVN copy number variant and/or internal tandem duplication (ITD). (PDF) [file pone.0318300.s001.pdf]

**Tursky M. L. *et al* . “Error-corrected ultradeep next-generation sequencing for detection of clonal haematopoiesis and haematological neoplasms – sensitivity, specificity and accuracy”.**

**S1 Table: Gene coverage of the Invitae VariantPlex Myeloid targeted NGS assay**. Shown are variant type, accession number and exons detected. SNV single nucleotide variant, indel insertion or deletion, CVN copy number variant and/or internal tandem duplication (ITD).

| Target | Variant | Accession | Exon |
|---|---|---|---|
| ABL1 | SNV/Indel | NM_005157 | 4-10 |
| ANKRD26 | SNV/Indel | NM_014915 | 1 (c.-113-c.-134) |
| ASXL1 | SNV/Indel, CNV | NM_015338.5 | 1-13 |
| | | NM_001164603.1 | 5 |
| ATRX | SNV/Indel | NM_000489 | 8-11,17-32 |
| BCOR | SNV/Indel, CNV, ITD | NM_017745 | 2-7,9-15 |
| | | NM_001123385 | 8 |
| BCORL1 | SNV/Indel | NM_021946 | 1-12 |
| BRAF | SNV/Indel | NM_004333 | 3,10-13,15 |
| BTK | SNV/Indel | NM_000061 | 15 |
| CALR | SNV/Indel | NM_004343 | 8,9 |
| CBL | SNV/Indel, CNV | NM_005188 | 2-5,7-9,16 |
| CBLB | SNV/Indel | NM_170662 | 3,9,10 |
| CBLC | SNV/Indel | NM_012116 | 9,10 |
| CCND2 | SNV/Indel | NM_001759 | 5 |
| CDC25C | CNV | | |
| CDKN2A | SNV/Indel, CNV | NM_058197 | 1 |
| | | NM_058195 | 1 |
| | | NM_000077 | 2,3 |
| | | NM_001195132 | 3 |
| CEBPA | SNV/Indel | NM_004364 | 1 |
| CSF3R | SNV/Indel | NM_156039 | 17 |
| | | NM_172313 | 10,18 |
| | | NM_000760 | 14-16 |
| CUX1 | SNV/Indel, CNV | NM_001202543 | 15-24 |
| | | NM_001913 | 1-23 |
| | | NM_181552 | 1 |
| CXCR4 | SNV/Indel | NM_003467 | 1,2 |
| DCK | SNV/Indel | NM_000788 | 2,3 |
| DDX41 | SNV/Indel | NM_016222 | 1-17 |
| DHX15 | SNV/Indel | NM_001358 | 3 |
| DNMT3A | SNV/Indel | NM_022552 | 2,3,5-23 |
| | | NM_153759 | 1,2 |
| | | NM_175630 | 4 |
| ETNK1 | SNV/Indel | NM_018638 | 3 |
| ETV6 | SNV/Indel, CNV | NM_001987 | 1-8 |
| EZH2 | SNV/Indel, CNV | NM_004456 | 2-20 |
| FBXW7 | SNV/Indel | NM_018315 | 1-11 |
| FLT3 | SNV/Indel, CNV, ITD | NM_004119 | 8-17,19-21 |
| GATA1 | SNV/Indel | NM_002049 | 2 |
| GATA2 | SNV/Indel | NM_032638 | 2-6 |
| GNAS | SNV/Indel | NM_000516 | 8-11 |
| HRAS | SNV/Indel | NM_005343 | 2-4 |
| IDH1 | SNV/Indel | NM_005896 | 3,4 |
| IDH2 | SNV/Indel | NM_002168 | 4,6 |
| IKZF1 | SNV/Indel, CNV | NM_001220769 | 5 |
| | | NM_001220767 | 2-5,7 |
| | | NM_001220771 | 4 |
| | | NM_001291845 | 4 |
| | | NM_001291847 | 5 |
| JAK2 | SNV/Indel | NM_004972 | 12-16,19-25 |
| JAK3 | SNV/Indel | NM_000215 | 3,11,13,15,18,19 |
| KDM6A | SNV/Indel, CNV | NM_021140 | 1-29 |
| | | NM_001291415 | 14 |
| KIT | SNV/Indel | NM_000222 | 1,2,5,8-15,17,18 |
| KMT2A | SNV/Indel | NM_005933 | 1-13,15-36 |
| | | NM_001197104 | 14 |
| KRAS | SNV/Indel | NM_004985 | 2-4 |
| LUC7L2 | SNV/Indel, CNV | NM_016019 | 1-10 |
| | | NM_001244585 | 2 |
| MAP2K1 | SNV/Indel | NM_002755 | 2,3 |
| MPL | SNV/Indel | NM_005373 | 10,12 |
| MYC | SNV/Indel, CNV | NM_002467 | 1-3 |
| MYD88 | SNV/Indel | NM_002468 | 4,5 |
| | | NM_001172567 | 3 |
| NF1 | SNV/Indel, CNV | NM_000267 | 1-14,16-57 |
| | | NM_001128147 | 15 |
| | | NM_001042492 | 31 |
| NOTCH1 | SNV/Indel | NM_017617 | 26-28,34,c.*370 to c.*380 |
| NPM1 | SNV/Indel | NM_002520 | 11 |
| NRAS | SNV/Indel | NM_002524 | 2-5 |
| PDGFRA | SNV/Indel | NM_006206 | 12,14,15,18 |
| PHF6 | SNV/Indel | NM_032335 | 2-8 |
| | | NM_001015877 | 10 |
| | | NM_032458 | 9 |
| PPM1D | SNV/Indel | NM_003620 | 6 |
| PTEN | SNV/Indel | NM_000314 | 1-9 |
| PTPN11 | SNV/Indel | NM_002834 | 3,4,7,8,12,13 |
| | | NM_080601 | 11 |
| RAD21 | SNV/Indel, CNV | NM_006265 | 2-14 |
| RBBP6 | SNV/Indel | NM_006910 | p.1444,p.1451,p.1569,p.1654,p.1673 |
| RPS14 | CNV | | |
| RUNX1 | SNV/Indel, CNV | NM_001754 | 2,3,5-9 |
| | | NM_001122607 | 1,5 |
| SETBP1 | SNV/Indel | NM_015559 | 4 (p.799-p.950) |
| SF3B1 | SNV/Indel | NM_012433 | 13-21 |
| SH2B3 | SNV/Indel | NM_005475 | 2-8 |
| SLC29A1 | SNV/Indel | NM_001078175 | 4,13 |
| SMC1A | SNV/Indel | NM_006306 | 1-25 |
| | | NM_001281463 | 2 |
| SMC3 | SNV/Indel | NM_005445 | 10,13,19,23,25,28 |
| SRSF2 | SNV/Indel | NM_003016 | 1,2 |
| STAG2 | SNV/Indel | NM_006603 | 2-33 |
| | | NM_001042749 | 32 |
| STAT3 | SNV/Indel | NM_003150 | 20 |
| | | NM_139276 | 21 |
| TET2 | SNV/Indel, CNV | NM_001127208 | 4-11 |
| | | NM_017628 | 3 |
| TP53 | SNV/Indel, CNV | NM_000546 | 1-11 |
| | | NM_001276696 | 10 |
| | | NM_001276695 | 10 |
| U2AF1 | SNV/Indel, CNV | NM_006758 | 2,6,7 |
| | | NM_001025204 | 6 |
| U2AF2 | SNV/Indel | NM_007279 | 1-12 |
| WT1 | SNV/Indel, CNV | NM_000378 | 1-7,9 |
| | | NM_001198552 | 8 |
| XPO1 | SNV/Indel | NM_003400 | 15,16,18 |
| ZRSR2 | SNV/Indel, CNV | NM_005089 | 1-11 |
