# Supplementary material for: Error-corrected ultradeep next-generation sequencing for detection of clonal haematopoiesis and haematological neoplasms – sensitivity, specificity and accuracy
Source: PLoS One. 2025 Feb 26;20(2):e0318300. doi: 10.1371/journal.pone.0318300 (PMC11864513; doi:10.1371/journal.pone.0318300)
Supplement: S2 Table — AML acute myeloid leukaemia, MDS myelodysplastic syndrome, MPN myeloproliferative neoplasm. * At the time testing by the reference laboratory the panels covering 7 or 26 genes used a 0.05-0.10 LLOD at a target read depth of 1000x for the majority of variants, an LLOD of 0.01 for JAK2:c.1849G > T and KIT:c.2447A > T and assessed ASXL1:c.1934dup by fragment analysis with an LLOD of 0.10. **The reference laboratory panel covering 41 genes used an LLOD of 0.02 and a target read depth of 500x for the most variants, with CEBPA LLOD ~ 0.10, ASXL1:c.1934dup LLOD ~ 0.05, and a stated coefficient of variance (CV) at 0.05 VAF of 0.102. (PDF) [file pone.0318300.s002.pdf]

Tursky M. L. *et al* . "Error-corrected ultradeep next-generation sequencing for detection of clonal haematopoiesis and haematological neoplasms – sensitivity, specificity and accuracy".

S2 Table: Reference laboratory samples and variants. Grouped as those with reported variants and those without, followed by participant diagnosis, showing the number of participants per group, number of assessable genes on each panel, number of variants reported by the reference laboratory, lower limit of detection (LLOD) used at the time of reference laboratory testing, number of samples negative for all variants tested by the reference laboratory. AML acute myeloid leukaemia, MDS myelodysplastic syndrome, MPN myeloproliferative neoplasm. *At the time testing by the reference laboratory the panels covering 7 or 26 genes used a 0.05-0.10 LLOD at a target read depth of 1000x for the majority of variants, an LLOD of 0.01 for JAK2:c.1849G>T and KIT:c.2447A>T and assessed ASXL1:c.1934dup by fragment analysis with an LLOD of 0.10. **The reference laboratory panel covering 41 genes used an LLOD of 0.02 and a target read depth of 500x for the most variants, with CEBPA LLOD ~0.10, ASXL1:c.1934dup LLOD ~0.05, and a stated coefficient of variance (CV) at 0.05 VAF of 0.102.

Category	Diagnosis	Number of participants	Number of assessable genes on panel	Number of variants reported	Stated lower limit of detection (LLOD) at time of testing	Number of samples negative for all variants tested
Reported variants	AML	7	26	21	0.05-0.10*	-
	MDS	4	26	8	0.05-0.10*	-
	MPN/MDS	1	26	5	0.05-0.10*	-
	MPN	4	7	2	0.05-0.10*	-
No reported variants	Cytopenia	2	26	-	0.05-0.10*	2
	MDS	1	41	-	0.02**	1
TOTAL	-	19	-	36	-	3

* Depth 1000x; exceptions JAK2 Val617Phe and KIT Asp816Val 0.01, ASXL1 c.1934dup performed with fragment analysis, limit 0.1.

** Depth 500x; exceptions CEBPA ~0.1, ASXL1 c.1934dup ~0.05. CV at 0.05 VAF = 0.102.
