# Supplementary material for: Error-corrected ultradeep next-generation sequencing for detection of clonal haematopoiesis and haematological neoplasms – sensitivity, specificity and accuracy
Source: PLoS One. 2025 Feb 26;20(2):e0318300. doi: 10.1371/journal.pone.0318300 (PMC11864513; doi:10.1371/journal.pone.0318300)
Supplement: S3 Table — Grouped by diagnosis, showing variant, average observed VAF, depth and UAO. AML acute myeloid leukaemia, MDS myelodysplastic syndrome, MPN myeloproliferative neoplasm. (PDF) [file pone.0318300.s003.pdf]

Tursky M. L. *et al* . "Error-corrected ultradeep next-generation sequencing for detection of clonal haematopoiesis and haematological neoplasms – sensitivity, specificity and accuracy".

S3 Table: Observed variants matching Reference laboratory reported variants. Grouped by diagnosis, showing variant, average observed VAF, depth and UAO. AML acute myeloid leukaemia, MDS myelodysplastic syndrome, MPN myeloproliferative neoplasm.

Diagnosis	Gene	Variant	Accession	Observed VAF	Depth x	UAO
AML	ASXL1	c.1900_1922del	NM_015338.5	0.3606	2654	268
	ASXL1	c.1934dup	NM_015338.5	0.2576	4636	350
	CEBPA	c.577_579dup	NM_001285829.1	0.4682	7266	546
	CEBPA	c.388_392dup	NM_001287424.1	0.4203	7881	533
	DNMT3A	c.2188C>A	NM_001320893.1	0.3047	844	141
	EZH2	c.165C>G	NM_001203247.1	0.4816	814	138
	EZH2	c.196C>T	NM_001203247.1	0.2104	2043	160
	FLT3	c.1775T>A	NM_004119.2	0.2377	7593	417
	FLT3	c.2504A>T	NM_004119.2	0.0491	5628	134
	FLT3-ITD	c.1770_1793dup	NM_004119.2	0.3362	3240	263
	GATA2	c.1114G>A	NM_001145661.1	0.0358	2070	50
	IDH2	c.263G>A	NM_001289910.1	0.4659	1732	255
	IDH2	c.359G>A	NM_001289910.1	0.1713	572	71
	KRAS	c.38G>A	NM_004985.3	0.1024	1104	69
	NPM1	c.860_863dup	NM_002520.6	0.2216	332	48
	RUNX1	c.101del	NM_001001890.2	0.0553	777	40
	RUNX1	c.181G>T	NM_001001890.2	0.1996	4354	289
	RUNX1	c.430A>G	NM_001001890.2	0.4655	4265	389
	RUNX1	c.512A>G	NM_001001890.2	0.9591	2545	520
	SRSF2	c.284_307del	NM_001195427.1	0.5614	1289	190
	SRSF2	c.284C>T	NM_001195427.1	0.4872	899	116
MDS	ASXL1	c.1782C>A	NM_015338.5	0.3636	1331	182
	ASXL1	c.1934dup	NM_015338.5	0.2155	2479	233
	ASXL1	c.2443_2444ins	NM_015338.5	0.0279	1324	16
	DNMT3A	c.2188C>T	NM_001320893.1	0.4045	927	164
	RUNX1	c.257C>T	NM_001001890.2	0.4329	961	134
	SF3B1	c.2098A>G	NM_012433.2	0.4734	1242	200
	SRSF2	c.284C>A	NM_001195427.1	0.4199	543	91
	TP53	c.824G>A	NM_000546.5	0.4546	1410	220
MPN/MDS	ASXL1	c.1900_1922del	NM_015338.5	0.0449	1537	60
	ASXL1	c.1933_1934del	NM_015338.5	0.0097	1643	15
	ASXL1	c.2839G>T	NM_015338.5	0.0288	902	22
	JAK2	c.1849G>T	NM_001322194.1	0.1206	829	68
	SF3B1	c.1996A>C	NM_012433.2	0.1855	760	90
MPN	CALR	c.1099_1150del	NM_004343.3	0.4617	379	102
	JAK2	c.1849G>T	NM_001322194.1	0.4812	1067	202
