# Supplementary material for: Error-corrected ultradeep next-generation sequencing for detection of clonal haematopoiesis and haematological neoplasms – sensitivity, specificity and accuracy
Source: PLoS One. 2025 Feb 26;20(2):e0318300. doi: 10.1371/journal.pone.0318300 (PMC11864513; doi:10.1371/journal.pone.0318300)
Supplement: S4 Table — Showing assessable genes. (PDF) [file pone.0318300.s004.pdf]

Tursky M. L. *et al* . "Error-corrected ultradeep next-generation sequencing for detection of clonal haematopoiesis and haematological neoplasms – sensitivity, specificity and accuracy".

S4 Table: Reference laboratory panels. Showing assessable genes.

Assessable genes in each reference laboratory panel		
**26 genes**	**7 genes**	**41 genes**
ASXL1	ASXL1	ABL1
BRAF	CALR	ASXL1
CALR	CSF3R	BRAF
CBL	JAK2	BTK
CEBPA	KIT	CALR
CSF3R	MPL	CBL
DNMT3A	SF3B1	CEBPA
EZH2		CSF3R
FLT3		CXCR4
FLT3-ITD		DDX41
GATA2		DNMT3A
IDH1		ETNK1
IDH2		EZH2
JAK2		FLT3
JAK3		FLT3-ITD
KIT		GATA1
KRAS		GATA2
MPL		IDH1
NPM1		IDH2
NRAS		JAK2
RUNX1		JAK3
SETBP1		KIT
SF3B1		KRAS
SRSF2		MAP2K1
TET2		MPL
TP53		MYD88
		NOTCH1
		NPM1
		NRAS
		PFH6
		RUNX1
		SETBP1
		SF3B1
		SH2B3
		SRSF2
		STAT3
		TET2
		TP53
		U2AF1
		XPO1
		ZRSR2
