# Supplementary material for: Error-corrected ultradeep next-generation sequencing for detection of clonal haematopoiesis and haematological neoplasms – sensitivity, specificity and accuracy
Source: PLoS One. 2025 Feb 26;20(2):e0318300. doi: 10.1371/journal.pone.0318300 (PMC11864513; doi:10.1371/journal.pone.0318300)
Supplement: S5 Table — Part of FAMAS cohort, including any previous diagnosis of Type II Diabetes, cancer, ischemic heart disease, or stroke, and prevalence of clonal haematopoiesis at different clone sizes. (PDF) [file pone.0318300.s005.pdf]

Tursky M. L. *et al* . "Error-corrected ultradeep next-generation sequencing for detection of clonal haematopoiesis and haematological neoplasms – sensitivity, specificity and accuracy".

S5 Table: Clinical characteristics of 383 community-dwelling adult males. Part of FAMAS cohort, including any previous diagnosis of Type II Diabetes, cancer, ischemic heart disease, or stroke, and prevalence of clonal haematopoiesis at different clone sizes.

Clinical Variable	Group	#	%
Age	<40	46	12%
	40-49	78	20%
	50-59	73	19%
	60-69	83	22%
	70-79	83	22%
	≥80	20	5%
Gender	Male	383	100%
	Female	0	0%
BMI	<18.5	0	0%
	18.5-24.9	76	20%
	25-29.9	163	43%
	≥30	131	34%
	Unknown	13	3%
Type II Diabetes	No	231	60%
	Yes	46	12%
	Unknown	106	28%
Cancer	No	325	85%
	Yes	52	14%
	Unknown	6	2%
Ischemic Heart Hisease	No	0	0%
	Yes	56	15%
	Unknown	327	85%
Stroke	No	261	68%
	Yes	16	4%
	Unknown	106	28%
CH Clone Size	<2.0%	384	100%
	2.0-5.0%	120	31%
	>5.0%	36	9%
