# Supplementary material for: Error-corrected ultradeep next-generation sequencing for detection of clonal haematopoiesis and haematological neoplasms – sensitivity, specificity and accuracy
Source: PLoS One. 2025 Feb 26;20(2):e0318300. doi: 10.1371/journal.pone.0318300 (PMC11864513; doi:10.1371/journal.pone.0318300)
Supplement: S6 Table — Used to independently validate low VAF CHIP variants identified from a cohort of community-dwelling adults. (PDF) [file pone.0318300.s006.pdf]

Tursky M. L. *et al* . "Error-corrected ultradeep next-generation sequencing for detection of clonal haematopoiesis and haematological neoplasms – sensitivity, specificity and accuracy".

S6 Table: Custom TaqMan Assays (ThermoFisher Scientific) for droplet digital PCR (ddPCR). Used to independently validate low VAF CHIP variants identified from a cohort of community-dwelling adults.

Gene	CDS Variant	Assay ID	Sequence used for design
TET2	c.1027A_C_2	ANYMX43	

CCAAAGCCAGCTGCAGTGGTGAGTGAGGCCTGTGATGCTGATGATGCTGATAATGCCAGTAAACTAGCTGCAATGCTAAATACCTGTT
CCTTTCAGAAACCAGAACAACTACAACAACAAAAATCAGTTTTTGAGATATGCCCATCTCCTGCAGAAAATAACATCCAGGGA[A/C]CC
ACAAAGCTAGCGTCTGGTGAAGAATTCTGTTCAGGTTCCAGCAGCAATTTGCAAGCTCCTGGTGGCAGCTCTGAACGGTATTTAAAAC
AAAATGAAATGAATGGTGCTTACTTCAAGCAAAGCTCAGTGTTCACTAAGGATTCCTTTTCTGCCACTACCACACCACCACCACCATCA
CAATTGCTTCTTT

KDM6A	c.2061A_C	ANMGCJN	

GACCCTTTTCTGCAGGCCATGTTCCCTGTAGCACATCAAGAACGCTGGGAAGTACAGACACTATTTTGATAGGCAATAATCATATAACA
GGAAGTGGAAGTAATGGAAACGTGCCTTACCTGCAGCGAAACGCACTCACTCTACCTCATAACCGCACAAACCTGACCAGCAGCGCA
GAGGAGCCGTGGAAAAACCA[A/C]CTATCTAACTCCACTCAGGGGCTTCACAAAGGTCAGAGTTCACATTCGGCAGGTCCTAATGGTG
AACGACCTCTCTCTTCCACTGGGCCTTCCCAGCATCTCCAGGCAGCTGGCTCTGGTATTCAGAATCAGAACGGACATCCCACCCTGCCT
AGCAATTCAGTA

NOTCH1	c.7531A_C	ANNK44K	

TCCTCGCTGGTCCCACCCGTGACCGCAGCCCAGTTCCTGACGCCCCCCTCGCAGCACAGCTACTCCTCGCCTGTGGACAACACCCCCAG
CCACCAGCTACAGGTGCCTGAGCACCCCTTCCTC[A/C]CCCCGTCCCCTGAGTCCCCTGACCAGTGGTCCAGCTCGTCCCCGCATTCCAA
CGTCTCCGACTGGTCCGAGGGCGTCTCCAGCCCTCCCACCAGCATGCAGTCCCAGATCGCCCGCATTCCGGAGGCCTTCAAGTAAACG
GCGCGCCCCACGAGACCCCGGCTTCCTT

TET2	c.542T_C	ANPRYPH	

CAAGAAATTGAAACAAGACCAAAAGGCTAATGGAGAAAGACGTAACTTCGGGGTAAGCCAAGAAAGAAATCCAGGTGAAAGCAGTC
AACCAAATGTCTCCGATTTGAGTGATAAGAAAGAATCTGTGAGTTCTGTAGCCCAAGAAAATGCAGTTAAAGATTTCACCAGTTTTTCA
ACACATAACTGCAGTGGGCCTGAAAATCCAGAGCTTCAGA[T/C]TCTGAATGAGCAGGAGGGGAAAAGTGCTAATTACCATGACAAG
AACATTGTATTACTTAAAAACAAGGCAGTGCTAATGCCTAATGGTGCTACAGTTTCTGCCTCTTCCGTGGAACACACACATGGTGAACT
CCTGGAAAAAACACTGTCTCAATATTATCCAGATTGTGTTTCCATTGCGGTGCAGAAAACCACATCTCACATAAATGCCATTAAC

RAD21	c.556A_C_2	ANXG4J6	

CATCGATGTGGCCCAGCAGTTCAGCTTGAATCAGAGTAGAGTGGAAGAGATAACCATGAGAGAAGAAGTTGGGAACATCAGTATTTT
ACAAGAAAATGATTTTGGTGATTTTGGAATGGATGATCGTGAGATAATGAGAGAAGGCAGTGCTTTTGAGGATGACGACATGTTAGT
AAGC[A/C]CTACTACTTCTAACCTCCTATTAGAGTCTGAACAGAGCACCAGCAATCTGAATGAGAAAATTAACCATTTAGAATATGAAG
ATCAATATAAGGATGATAATTTTGGAGAAGGAAATGATGGTGGAATATTAGATGACAAACTTATTAGTAATAATGATGGCGGTATCTT
TGATGATCCCCC
