# Supplementary material for: Error-corrected ultradeep next-generation sequencing for detection of clonal haematopoiesis and haematological neoplasms – sensitivity, specificity and accuracy
Source: PLoS One. 2025 Feb 26;20(2):e0318300. doi: 10.1371/journal.pone.0318300 (PMC11864513; doi:10.1371/journal.pone.0318300)
Supplement: S7 Table — Comparison of parameters to sequence 144 samples at specific read depths of 500x, 1000x, 2000x, 3000x, 4000x and 5000x, shown relative to values for a depth of 3000x (indicated in bold). Time for bioinformatic analysis covers the pipeline from raw NGS data to output of a list of variants and does not include curation of this list. Lower read depths (500x) can be achieved by including a higher number of samples per run. This required the same cost and time per 144 samples for preparation and quantification, and resulted in an 0.8-fold lower total cost and 0.7 less total time due to the increased number of samples per run and smaller raw data files. Increasing depth, to enable accurate sequencing of very low VAF resulted in a 1.1 to 1.3-fold higher total cost and time requirement overall. Refer to S8 Table for original values for this assay in hours and Australian dollars. (PDF) [file pone.0318300.s007.pdf]

Tursky M. L. ***et al*** **. "Error-corrected ultradeep next-generation sequencing for detection of clonal haematopoiesis and haematological neoplasms – sensitivity, specificity and accuracy".**

S7 Table: **Impact of target read depth on cost and time shown relative to values for a depth of 3000x**. Comparison of parameters to sequence 144 samples at specific read depths of 500x, 1000x, 2000x, 3000x, 4000x and 5000x, shown relative to values for a depth of 3000x (indicated in bold). Time for bioinformatic analysis covers the pipeline from raw NGS data to output of a list of variants and does not include curation of this list. Lower read depths (500x) can be achieved by including a higher number of samples per run. This required the same cost and time per 144 samples for preparation and quantification, and resulted in an 0.8-fold lower total cost and 0.7 less total time due to the increased number of samples per run and smaller raw data files. Increasing depth, to enable accurate sequencing of very low VAF resulted in a 1.1 to 1.3-fold higher total cost and time requirement overall. Refer to S8 Table for original values for this assay in hours and Australian dollars.

Depth	Raw data file size (GB)	Cost library preparation and quantification	Cost of targeted panel sequencing	Cost of longterm storage of data files	TOTAL cost	Time for library prep and quantification	Time for sequencing	Time for bioinformatic analysis	TOTAL time
500x	0.2	1.0	0.3	0.2	0.8	1.0	0.3	0.2	0.7
1000x	0.3	1.0	0.4	0.3	0.8	1.0	0.4	0.3	0.8
2000x	0.7	1.0	0.7	0.7	0.9	1.0	0.7	0.7	0.9
**3000x**	**1**	**1**	**1**	**1**	**1**	**1**	**1**	**1**	**1**
4000x	1.3	1.0	1.3	1.3	1.1	1.0	1.3	1.3	1.1
5000x	1.7	1.0	1.8	1.7	1.3	1.0	1.8	1.7	1.3
