# Supplementary material for: Error-corrected ultradeep next-generation sequencing for detection of clonal haematopoiesis and haematological neoplasms – sensitivity, specificity and accuracy
Source: PLoS One. 2025 Feb 26;20(2):e0318300. doi: 10.1371/journal.pone.0318300 (PMC11864513; doi:10.1371/journal.pone.0318300)
Supplement: S8 Table — Raw data file sizes are in gigabytes (GB), costs are in Australian dollars (AUD) and time is indicated in hours. Time for bioinformatic analysis covers the pipeline from raw NGS data to output of a list of variants and does not include curation of this list. Lower read depths (500x) can be achieved by including higher number of samples per run, and results in smaller raw data file size per 144 samples. The cost and time for preparation and quantification per 144 samples remains the same. Cost of sequencing, data storage, time for sequencing and bioinformatics all vary by raw data file size, resulting in changes to total cost and time. Refer to S7 Table for these values shown relative to values at a read depth of 3000x (indicated in bold). (PDF) [file pone.0318300.s008.pdf]

Tursky M. L. *et al* . "Error-corrected ultradeep next-generation sequencing for detection of clonal haematopoiesis and haematological neoplasms – sensitivity, specificity and accuracy".

S8 Table: Comparison of cost and time parameters to sequence 144 samples at specific read depths. Raw data file sizes are in gigabytes (GB), costs are in Australian dollars (AUD) and time is indicated in hours. Time for bioinformatic analysis covers the pipeline from raw NGS data to output of a list of variants and does not include curation of this list. Lower read depths (500x) can be achieved by including higher number of samples per run, and results in smaller raw data file size per 144 samples. The cost and time for preparation and quantification per 144 samples remains the same. Cost of sequencing, data storage, time for sequencing and bioinformatics all vary by raw data file size, resulting in changes to total cost and time. Refer to S7 Table for these values shown relative to values at a read depth of 3000x (indicated in bold).

Depth	Raw data file size (GB)	Cost library preparation and quantification (AUD)	Cost of targeted panel sequencing (AUD)	Cost of longterm storage of data files (AUD)	TOTAL cost (AUD)	Time for library prep and quantification (Hours)	Time for sequencing (Hours)	Time for bioinformatic analysis (Hours)	TOTAL time (Hours)
500x	300	$14,040	$2,089	$43	$16,172	130	13	5	148
1000x	600	$14,040	$3,133	$86	$17,259	130	20	10	160
2000x	1200	$14,040	$4,700	$172	$18,912	130	29	19	178
**3000x**	**1800**	**$14,040**	**$7,050**	**$258**	**$21,348**	**130**	**44**	**29**	**203**
4000x	2400	$14,040	$9,400	$344	$23,784	130	59	39	228
5000x	3000	$14,040	$12,533	$430	$27,003	130	78	48	256
